# Solutions for frontier AI safety: the case for science-driven international cooperation and standardization at a time of radical transformations

**DOI:** 10.3389/frai.2026.1950290

**Published:** 2026-10-27

**Authors:** Daniele Gerundino, Yishu Mao, Leonardo Chiariglione, Zheng Liang

**Affiliations:** 1 University of Geneva, Geneva, Switzerland; 2 Institute of Management, Scuola Superiore di St. Anna, Pisa, Italy; 3 Department of Communication and Media Research, University of Zurich, Zürich, Switzerland; 4 Max Planck Institute for the History of Science, Lise Meitner Research Group: China in the Global System of Science, Berlin, Germany; 5 Humboldt University of Berlin, The Institute for Asian and African Studies, Berlin, Germany; 6 Department of Communications and Media Research, University of Zurich, Zurich, Switzerland; 7 Moving Picture, Audio and Data Coding by Artificial Intelligence, Geneva, Switzerland; 8 School of Public Policy and Management, Tsinghua University, Beijing, China; 9 Institute for AI International Governance of Tsinghua University (I-AIIG), Beijing, China; 10 China Institute for Science and Technology Policy at Tsinghua University (CISTP), Beijing, China

**Keywords:** artificial intelligence, historical example, international collaboration, safety, standard

## Abstract

As AI systems and their underlying technologies continue to advance at extraordinary speed, eminent scientists are warning of risks, including extreme risks. While high-profile governmental and intergovernmental initiatives such as AI summits, OECD and UN initiatives have raised hopes, substantial gaps remain between the severity of potential harms and the capacity of these initiatives to address them. Owing to limited research, private sector dominance, minimal investment in AI safety, the voluntary nature of most measures recommended, and the current geopolitical context, we argue that it is unlikely that multilateral actions will deliver the effective and timely measures required. Other approaches are needed and are possible. They should not be regarded as alternatives to multilateral efforts, but more as complementary actions to provide concrete and timely solutions that, at some point, can be integrated into the multilateral framework. We take inspiration from historical examples of scientific, technical and organizational collaboration in which a plurality of committed entities has achieved outstanding results. These historical examples show that, even with limited resources, collaboration can enormously increase the collective ability to attain far-reaching goals. We propose the creation of an international network led by top scientists and composed of AI labs and research groups, think tanks and other organizations keen to share ideas, materials and data, while pursuing three common goals: (1) advance AI safety science; (2) develop practical techniques, tools, best practices, and technologies for building safe AI systems; (3) create standards for operationalizing the use of these tools and technologies, including conformity assessment mechanisms, to support their widespread adoption.

## 1 Introduction

The extraordinary development of artificial intelligence systems that has occurred over the past decade, and notably after the release of transformer architecture (Vaswani et al., 2017) and Chat GPT (OpenAI, 2022), is having an enormous impact on science and technology, on the economy, on society, as well as on the future of humanity. The perception of the transformative potential of AI, combined with the phenomenal pace of its development, has also generated a growing awareness of AI risks among the scientific community, the experts involved in its development, policy makers, and a plurality of other stakeholders.

In this context, efforts have been made by some governments, international organizations and other actors to address AI risks. In the following sections, we provide an overview of some of them. However, we believe it is reasonably evident that: existing efforts so far are insufficient to satisfactorily mitigate such risks, particularly those that are considered “extreme risks” (Bengio et al., 2023) related to frontier models (Bullock et al., 2024).

The primary objective of this paper is therefore a call to action that offers a new possible approach for managing AI risks: a science-driven and focused international cooperation. This should not be considered “alternative” to current and possible future multilateral efforts, but complementary, as a way to develop concrete and effective solutions in a short timeframe.

For this reason, Section 2 of the paper is dedicated to the proposal outline—with the understanding that at this stage, as a call to action, it should not be regarded as an articulated and detailed project plan.

The following Section 3 provides information on selected important initiatives undertaken by governments and international organizations to address AI risks (or at least certain types of AI risks). It also highlights how, from a high-level perspective, considering the industrial and geopolitical context, along with recent incidents and trends, we cannot reasonably expect adequate and timely protection from these initiatives.

Section 4 provides a rationale for science-driven international cooperation in AI safety. It also highlights outstanding historical examples of science-driven international projects that have achieved far-reaching goals, from which it is possible to derive inspiration and insights. Finally, it considers key challenges concerning the implementation of our proposal, and how to address them.

The last Section 5 provides a summary of the goal of the proposal, considers possible objections and indicates directions for future research.

## 2 Our proposal

### 2.1 A science-driven international cooperation for AI safety

We call for science-driven international cooperation in frontier AI safety governance through the creation of a dedicated international network or consortium focused on AI safety (hereafter, the Network), run by leading scientists from relevant disciplines (involving the research entities where they work) and operating independently of formal national affiliations. Additionally, we propose a set of strategic goals for the Network and outline the core elements of a feasible implementation strategy.

We wish to emphasize that this proposal is complementary to initiatives pursued by existing networks and the current multilateral system. Meanwhile, it proposes to go beyond the current efforts by establishing a structured and sustained collaboration, pursuing common goals through specific projects, and by seeking concrete and timely outputs. This approach is similar to that practiced to great effect in the historical examples outlined in later sections.

The Network can be established by a core group of founders, and its activities can be implemented in steps, for example, starting with one or a few projects, allowing its members to experiment with modes of collaboration and other important aspects. For instance, one promising initial area of activity could be the development of an index or measurement framework to track frontier AI capabilities and risks, or the development of benchmarks and testing for the evaluation, verification and validation of AI models, systems and agents’ safety against agreed criteria.

### 2.2 Strategic goals of the network

1. Advance and coordinate AI safety science: The Network should engage in coordinated research aimed at advancing the scientific foundations of the emerging field of AI safety. This includes i.a. matters such as in-depth analyses and characterization of harms associated with general-purpose AI, understanding and explaining why and how AI systems might exhibit and engage in harmful behaviors, the development of methods and techniques aiming to prevent or mitigate the effect of such behaviors, the development of benchmarks and testing for the evaluation, verification and validation of AI models, systems and agents’ safety against agreed criteria.
2. Develop practical techniques, tools, best practices, and technologies for designing and building safe AI systems: Building on, and in parallel with advances in AI safety science pursued under Goal 1, the Network should develop techniques, tools and services that can be applied throughout the AI lifecycle. These may include protocols, audit and evaluation techniques, benchmarks, good practices and procedures, software implementations and APIs, specialized hardware solutions (such as hardware-enabled governance mechanisms, HEMs), as well as datasets, test suites, and monitoring tools.
3. Develop standards and conformity assessment mechanisms: The Network should translate the outputs of Goals 1 and 2 into draft standards that operationalize AI safety in practice, including specifications, documentation of procedures and good practices, criteria and thresholds, and test methods—potentially supported by AI-enabled software suites. Standards development activities should be closely integrated with the technology-development cycle to shorten timelines and ensure practical relevance. These deliverables would function as “pre-standards,” to be submitted to formal international standardization bodies for review, refinement and possible adoption.

To maximize dissemination and uptake, most outputs—particularly tools and compliance assessment mechanisms—should be released as open source, with carefully designed safeguards to prevent fragmentation or the emergence of proprietary forks that could undermine the ecosystem.

### 2.3 Core elements of a feasible implementation strategy

The success of the proposed Network depends on striking a healthy balance between effective coordination and decentralized research activity. While recognizing the significant challenges involved, we outline a few core elements of a feasible implementation strategy.

1. Convening a core scientific community: Identify an initial group of leading scientists and research institutes across relevant disciplines and host dedicated workshops and focused exchanges, to define the objectives, scope, and priorities of the Network. This process should build on existing efforts, such as the International AI Safety Report and the Singapore Consensus, and on concrete indications from International AI safety summits and other key initiatives. In this respect, the International Association for Safe & Ethical AI (IASEAI), set up in 2024 “to unite the efforts of hundreds of organizations and thousands of individuals concerned with safe and ethical AI”, could play a very important role, contributing to the offspring, from the community they represent (mostly dedicated to advocacy and information sharing), of a project-based, outcome-oriented entity.
2. Creating light but effective governance structures: Establish a founding board and a small office responsible for coordination, communication and stewardship of shared resources, while avoiding excessive bureaucratic overheads.
3. Securing mission-aligned funding: Engage potential donors—initially private and non-profit organizations with established interests in AI safety, ethics, alignment, or related areas—to present the initiatives, gather feedback, and refine its governance model, funding structure, timelines, resource allocation and licensing frameworks. While public funding would be welcome, the autonomous nature of identifying research priorities and reaching consensus among scientists should be preserved.
4. Launching collaborative project teams: Form project-based teams involving multiple organizations, each with designated coordinators, clear objectives and timelines. Collaboration could be primarily open source based, and could include explicit decisions on licensing, governance, infrastructure and contribution modalities. While most projects should follow open collaboration models, more restrictive arrangements might be warranted in selected, highly sensitive areas.
5. Ensuring rapid knowledge exchange: Establish agile mechanisms to share interim results, solicit feedback and integrate contributions from across the network, and, where appropriate, from the broader research community to accelerate learning and convergence.

## 3 Existing intergovernmental initiatives and conventional standardization processes vs. AI capability acceleration: why this network is needed

### 3.1 Existing intergovernmental initiatives and conventional standardization processes

The breakneck pace of AI development and its rapid diffusion across nearly all domains of life have brought ethics, regulation and safety to the center of global attention. Because the impacts of advanced AI systems are not limited to national borders, the need for international cooperation is widely acknowledged. In recent years, governments and intergovernmental organizations have launched a growing number of actions.

A selection of key initiatives and documents is presented below. It includes examples of voluntary measures and legislation from important jurisdictions, principles, resources and actions from international organizations, and directions from government-led AI summits. This reflects the thoughts of the authors and there is no pretense of exhaustivity.

1. The Risk Management Framework (RMF) (NIST AI RMF 1.0, 2021) developed by NIST, the US National Institute of Standards and Technology (a non-regulatory federal agency within the U.S. Department of Commerce).

The RMF provides a framework (including concepts, methods, tools and guidance) to help managing risks to individuals, organizations, and society associated with artificial intelligence. The RMF, released in January 2023, was developed through a consensus-driven process involving a plurality of stakeholders and is intended for voluntary use,

NIST’s RMF recognizes that “the risks posed by AI systems are in many ways unique. AI systems, for example, may be trained on data that can change over time, sometimes significantly and unexpectedly, affecting system functionality and trustworthiness in ways that are hard to understand. AI systems and the contexts in which they are deployed are frequently complex, making it difficult to detect and respond to failures when they occur. AI systems are inherently socio-technical in nature, meaning they are influenced by societal dynamics and human behavior. […] These risks make AI a uniquely challenging technology to deploy and utilize both for organizations and within society. […] The Framework is designed to equip organizations and individuals—referred to here as AI actors—with approaches that increase the trustworthiness of AI systems, and to help foster the responsible design, development, deployment, and use of AI systems over time” (Executive summary of NIST RMF).

The RMF is complemented by a variety of additional resources, including notably NIST-AI-600-1, Artificial Intelligence Risk Management Framework: Generative Artificial Intelligence Profile, released in July 2024 (Autio et al., 2024), and a concept note for an AI RMF Profile on Trustworthy AI in Critical Infrastructure, released in April 2026 (Sheh and Stanley, 2026).

1. EU AI Act, the first comprehensive AI regulatory framework (EU AI Act, 2024).

Developed through an open, consensus-driven process started in 2018, the AI Act provides a common legal framework for the European Union for AI systems, following the customary EU risk-based approach for product safety: regulatory requirements are posed on providers and distributors of AI systems intended for professional use (systems used for military, national security and research purposes are exempted). The AI Act defines Risk (Art. 3(2)) as “The combination of the probability of an occurrence of harm and the severity of that harm.” and establishes four categories of risk: “Unacceptable risk” (AI systems in this category are prohibited), “High-risk” (AI systems in this category are deemed to have a significant potential impact on health, safety and fundamental rights, and most of the regulatory requirements of the AI Act concern them), “Limited/transparency risk” (with straightforward “transparency” obligations), and “Minimal risk” (systems in this category are not regulated). Risks belonging to the four categories are explicitly described considering their potential impact on citizens in general (e.g., systems deploying subliminal, manipulative, or deceptive techniques to distort behavior) or because they affect specific stakeholder groups (e.g., systems exploiting vulnerabilities related to age, disability, or socio-economic circumstances to distort behavior), combined in some cases with specific technical features (e.g., biometric categorization systems inferring sensitive attributes).

The AI risks identified and addressed in the AI Act are treated similarly to other types of risks associated with “conventional” digital technologies. They concern systems’ behavior and outcomes for specific activities regarding given target groups. However, due to the advent and importance of generative AI, specific requirements have been included (in 2023) for such systems, which are called “General Purpose AI (GPAI)” in the AI act. The EU Artificial Intelligence Act entered into force on 1 August 2024, including its Chapter V, which outlines the obligations for GPAI models—the specific provisions for GPAIs were implemented on 2 August 2025, and the European Commission’s enforcement and supervision powers for GPAI came into force on 2 August 2026 (EU, 2026).^1^

1. The Interim Measures for the Administration of Generative AI Services China (China Law Translate, 2023).

To date, this is the most significant Chinese regulation covering generative AI. It was promulgated by the Cyberspace Administration of China (CAC),^2^ jointly with other six Chinese ministries, in July 2023 and entered into force on 15 August 2023. The Measures apply to services that use generative AI technology to provide generated content (such as text, images, video and audio) to the public within the territory of the People’s Republic of China, including AI services through programmable APIs.

The measures follow a principles-based approach: balancing innovation and security, upholding core socialist values, respecting social morality and ethics, and not generating content that incites the subversion of state power. They also pose operational obligations on providers in several interrelated areas: Data legality and provenance, Personal information protection, Content safety and management, Labeling, Risk mitigation, and Accountability.

The measures require the AI service providers concerned to fulfil several obligations (set up adequate organizational structures, adopt data governance, perform risk assessment and implement technical mitigation measures) and undergo pre-deployment security assessments, submit mandatory documentation to CAC and complete the official algorithmic filing system registration. According to CAC, as of 30 June 2026, 988 generative AI services had completed filing and 598 applications/functions had completed registration (Cyberspace Administration of China (CAC), 2026).

It is important to note that the approach followed by China with respect to AI regulation, so far at least, is not based on a single, broad and overarching AI regulation, but on a vertical, layered set of legislative instruments covering different types of functionalities or application scenarios.

That said, in 2026, further to the rapid and pervasive dissemination of agents (and notably OpenClaw), the attention of policy makers has been strongly directed towards agents’ safety (shifting from content control to action control), with CAC and various other organizations taking actions to address the issue (Wagner et al., 2026).

1. Initiatives led and resources provided by the OECD. These include notably:

The OECD AI Principles, approved by the OECD Council meeting at the Ministerial level in May 2019 and amended in May 2024 (OECD, 2025). They comprise five high-level value-based principles—intended to “promote use of AI that is innovative and trustworthy and that respects human rights and democratic values.” —and five recommendations for national policies and international cooperation regarding AI.

The AI Policy Observatory (OECD, 2026a) and the informal OECD Network of Experts on AI (OECD, 2026b) launched in February 2020 to provide an inclusive forum for exchanging information on AI policy and activities, and to foster multi-stakeholder and interdisciplinary dialogue.

A framework for comparing tools and practices to implement trustworthy AI systems as set out in the OECD AI Principles (OECD, 2021).

Other important resources, such as the Catalogue of tools and metrics for trustworthy AI (OECD-GPAI, 2023) and the database (OECD, n.d.) launched after the merger in July 2024 with the Global Partnership on Artificial Intelligence (GPAI–an international, multistakeholder initiative), strive to promote the responsible development and use of artificial intelligence.

1. Initiatives led and resources provided by UNESCO (a specialized agency of the United Nations that seeks to build peace and security worldwide through international cooperation in education, science, culture, and communication).

A key reference is the Recommendation on the Ethics of Artificial Intelligence, published by UNESCO (2026) and adopted by 193 member states. Central to the Recommendation are a set of core values, providing the foundation for a Human-rights based approach to AI. The Recommendation also set forth ten core principles that should guide AI Ethics at all levels (Proportionality and Do No Harm, Safety and Security, Responsibility and Accountability; Transparency and Explainability; Human Oversight and Determination; Fairness and Non-Discrimination; Right to Privacy and Data Protection; Multi-stakeholder and Adaptive Governance and Collaboration; Sustainability; Awareness and Literacy.)

The Recommendation also provides guidance on 11 key areas for policy action that member states should undertake to pursue responsible development of AI. To support the implementation of the Recommendation, UNESCO has also made available methodologies and tools such as the Readiness Assessment Methodology and the Ethical Impact Assessment.

UNESCO has subsequently launched, in 2024 (in partnership with ITU and the Turing Institute), the Global AI Ethics and Governance Observatory (UNESCO, 2026) a repository of information and resources aiming at guiding policymakers, regulators, and other stakeholders to address ethical and governance aspects of AI.

1. Activities covered by the United Nations (in general).

The United Nations started to consider issues related to AI in 2015 and its activities in this area can roughly be divided into three main streams:

- Lethal Autonomous Weapons (LAWS), a subject addressed by several UN entities (including the General Assembly), with the UN Secretary-General, António Guterres, and several prominent international leaders, advocating for a new international treaty setting out specific prohibitions and restrictions on LAWS.
- AI supporting the 2030 Agenda for sustainable development (SDGs)–a theme covered by a broad variety of UN agencies and other entities, all involved in the network “AI for Good”, launched in 2017 and coordinated by the International Telecommunication Union (ITU).
- AI Ethics and Global AI Governance. In this area, along with the work of UNESCO on AI Ethics, the focus of the UN system’s actions concerns directly AI governance. The UN Secretary-General’s High-level Advisory Body on Artificial Intelligence was proposed in 2020 and set up in October 2023. After extensive consultations worldwide involving a large number of experts in different fields, it released its final report, “Governing AI for Humanity” (United Nations, 2024), in September 2024. The Report advocates the development of global, inclusive and distributed architecture for AI governance based on international cooperation. It identifies the main gaps in the existing International AI governance framework and provides seven main recommendations to address them.

Following the release of Governing AI for Humanity, intergovernmental negotiations and broad consultations with diverse stakeholders, the United Nations General Assembly established the Independent International Scientific Panel on Artificial Intelligence and the Global Dialogue on Artificial Intelligence Governance on 26 August 2025. The Preliminary Report of the 2026 Independent International Scientific Panel on AI that included evidence-based assessment of opportunities, risks and impacts of AI and an independent scientific assessment of the capabilities, emerging opportunities and risks of artificial intelligence was released on 1 July 2026 and subsequently presented and discussed during the first session of the AI Global Dialogue on AI Governance, convened in Geneva (CH) on 6–7 July 2026 (Independent International Scientific Panel on AI, 2026).

1. Government-led AI Summits.

The first one, the AI Safety Summit, was organized by the UK government at Bletchley Park (UK) on 1–2 November 2023.

The UK summit brought together 28 countries including the US, China and the EU, with over 150 attendees representing national governments, industry, academia, and civil society.

The UK government highlighted two key concerns, the risks of misuse and the risks of loss-of-control, and suggested prioritizing attention on frontier AI, which was defined (in documentation released in advance of the summit) as “highly capable general-purpose AI models that can perform a wide variety of tasks and match or exceed the capabilities present in today’s most advanced models.”

The summit recognized that frontier AI systems pose significant risks, including potential catastrophic harm. All 28 participating countries (and the EU) signed the “Bletchley Declaration” (GOV.UK, 2023), recognizing the urgency of addressing the risks posed by frontier AI, and committed to work together to define a global cooperative response.

Participants also agreed to conduct testing for frontier AI models, before and post deployment, against a variety of potential harms. In conjunction with the summit, the UK government announced the establishment of the UK AI Safety Institute (UK AISI). The US AI Safety Institute (US-AISI), under the responsibility of NIST, was also established in conjunction with the summit.

In May 2024, South Korea, in partnership with UK, co-hosted the AI Seoul Summit–as agreed at the previous UK summit. The Seoul summit showed the continued good intent of a number of actors, but also gave a clear idea of the complexity faced by global, multilateral initiatives in this field–something that is revealed by the more than six declarations/statements/pledges from different actors or groups of countries issued at the summit. It was also a bit confusing to have two different outcome documents signed by government representatives. The “Seoul Ministerial Statement for advancing AI safety, innovation and inclusivity: AI Seoul Summit 2024” (Ministry of Foreign Affairs, Republic of Korea, 2024), signed by 27 countries and the EU, and the “Seoul Declaration for Safe, Innovative and Inclusive AI,” signed by the governments of 10 countries and the EU. The Declaration includes an Annex “Seoul Statement of Intent toward International cooperation on AI Safety Science,” which i.a. expressed the “shared ambition to develop an international network among key partners to accelerate the advancement of the science of AI safety.” Following the Seoul summit, some of the signatory countries set up their national AI Safety Institutes and, in November 2024, the International Network of AI Safety Institutes was established.

However, to highlight the complexity of the process, the influence of geopolitical factors and the gap between aspirations and action, it should be noted that China did not sign the Seoul Ministerial Statement nor the Seoul Declaration and was not involved (at least formally) in the International Network of AI Safety Institutes.

The summits organized in the following years–the “AI Action Summit”, Paris, 10–11 February 2025 (Elysee, 2025) and the “AI Impact Summit”, Delhi, 16–20 February 2026 (MEA, 2026), while growing enormously in terms of audience and participation of political and business leaders, removed any direct reference to “AI safety” and oriented focus and priorities on other themes.

The last event outlined in these notes was not directly part of the sequence following the Bletchley Park summit. However, considering its particular relevance for the Asian region and the quality of the outcome, we have decided to include the Singapore Conference on AI (SCAI) that took place on 26 April 2025. SCAI 2025, “International Scientific Exchange on AI Safety”, strived to advance technically and scientifically grounded conversations on AI Safety (The Singapore Consensus, 2025).

### 3.2 AI capability acceleration: why this network is needed

As the above-mentioned initiatives unfolded over the past few years, frontier AI development has been accelerating, accompanied by claims that existential risks could emerge in a few years or even sooner. There is clear evidence that frontier models can already be directed to pursue malicious goals (Anthropic, 2025) and are not only capable of but also practice misaligned behaviors (Greenblatt et al., 2024), such as deception, jailbreaking, scheming, sandbagging and more (Meinke et al., 2024; Lynch et al., 2025).

In 2026 several striking incidents and emerging patterns have shown how serious and concrete these concerns are.

For example, an evident trend concerns the substantial and rapidly increasing capabilities of AI systems in cybersecurity. As a result, “the operational window between vulnerability disclosure and weaponized exploitation is collapsing. Once measured in months, this window has been compressed to days and, with increasing frequency, to hours. […] Artificial intelligence has become the primary force driving this compression. AI systems can now generate working proof-of-concept exploit code for published CVEs in as little as 10 to 15 minutes at a cost of approximately one dollar per attempt.” (Cloud Security Alliance AI Safety Initiative, 2026).

The announcement of Anthropic Mythos Preview on 7 April 2026 (Anthropic, 2026; UK AISI, 2026), the uproar it generated, and the questions and debates it triggered, are all part of this trend (Goldstein, 2026; Lee and Schneier, 2026; Osmond, 2026).

Another clear trend concerns the rapid development and dissemination of AI agents. From the perspective of AI safety, a major risk is represented by unsolicited actions of agents able to bypass boundaries, escape sandboxes and scheming (covertly pursuing misaligned goals). Incidents that made newspaper headlines in 2026 belong to this category: such as those concerning Alibaba agent’s ROME that engaged in unauthorized crypto mining and network tunneling in March 2026 (AIM AI Incidents and Hazards Monitor, 2026), or the autonomous AI agent driven by a combination of OpenAI models that, in July 2026, run an end-to-end intrusion against the Hugging Face platform to find and steal solutions and datasets for an advanced cybersecurity benchmark (Larcher et al., 2026), or the incident discovered by the UK AISI on 28 July 2026, where frontier’s AI models form Anthropic and Open AI created fake online identities and actions to socially pressure real human maintainers into approving malicious code for an open source project. According to AISI, “we found that some of the agents being tested had engaged in sustained, potentially harmful activity directed at real people and organizations” (UK AI Security Institute, 2026).

These incidents concerned systems undergoing security evaluations within test labs. However, they give a big warning signal: as the systems’ capabilities increase, the environments to safely test their limits, even those used by the organizations with the most expertise and resources, are failing to contain them (Bellan, 2026).

All this raises serious questions about whether current government-led coordination mechanisms are adequate. Under today’s geopolitical conditions, marked by the US’ rejection of AI international governance (Kratsios, 2025) and the strategic rivalry between the United States and China, intergovernmental initiatives face growing structural constraints. The post-war international order that once enabled a certain level of global governance of science and technology through organizations such as the International Atomic Energy Agency (IAEA) and the Biological Weapons Convention (BWC), no longer provides a stable foundation for consensus-building in fast-moving and disruptive fields such as AI.

These challenges are already visible. Despite the attention generated by the first AI summits, early ambitions for a coherent international “AI safety network” have weakened. In the United States and the United Kingdom, institutional priorities have shifted from safety, towards “AI security,” emphasizing threats posed by adversarial actors rather than systemic or unintended risks (UK DIST, 2025; Lutnick, 2025). Political turnover has further reduced continuity, as illustrated by the rollback of federal AI safety initiatives in the United States under Trump’s administration (Executive Orders, 2025) and by the fact that the EU AI Act—approved in 2024 and complemented by implementation measures in 2025—has been seriously challenged, and its implementation significantly delayed (Euronews, 2025). As outlined above, the shift of focus from AI safety to other matters in the global AI summits, are also clear illustrative signals of this broader trend.

Similar challenges can be observed in conventional standardization processes. Standardization, particularly at the international level, has long played a central role in promoting good practices by providing shared definitions, criteria, rules and guidelines for materials, products, systems and processes. Across diverse sectors, including chemicals, construction, electrotechnology, health care, transportation and information technology, voluntary standards have supported the development and functioning of markets, and have complemented regulations and helped operationalize regulatory objectives (Gerundino et al., 2024). Standards also underpin conformity assessment activities such as testing, inspection and certification, which are foundational to most regulatory regimes, with their stringency typically calibrated to the level of risk involved.

Not surprisingly, standardization and conformity assessment are explicitly or implicitly embedded in all major AI governance frameworks currently formulated or under development. ISO, IEC and ITU, the three most prominent international standardizing organizations, are all pursuing efforts to develop standards. However, under the current geopolitical and industrial conditions, it is unlikely that these conventional international standardization processes alone can deliver timely and robust solutions to the challenges posed by frontier AI. Two structural constraints are particularly salient. First, standards-setting processes are heavily influenced by leading industry actors who are simultaneously engaged in intense competition over AI capabilities. This creates persistent tensions between commercial and power incentives and the development of safety-oriented standards that could be perceived as constraining performance or market leadership. Second, while formal standardization procedures are designed to be inclusive, transparent and consensus-driven, they are inherently time-intensive. Even when accelerated, such processes struggle to keep up with the rapid and iterative development cycles characteristic of contemporary AI systems.

At the same time, empirical evidence indicates that AI capabilities are continuing to scale at an extraordinary pace. Among the many available sources, we refer to the analyses made by Epoch AI, a leading nonprofit research organization tracking long-term AI trends. Since 2010, the computational performance of machine-learning hardware has doubled approximately every 2.2 years, while the volume of training data used by AI models has doubled every eight to 9 months, with datasets for leading large language models (LLMs) tripling annually over the same period (Sevilla et al., 2022). Even more strikingly, the amount of computation required to train notable and frontier models has increased by roughly four to five times per year since 2010, with further acceleration in recent years, a trend that Epoch AI projects will continue through 2030 despite the need for unprecedented infrastructure and investment. While algorithmic progress is harder to measure (Brown et al., 2024), Epoch AI’s retrospective analysis of 2012–2023 shows that the compute required to reach a fixed performance threshold (a practical proxy for algorithmic efficiency) has halved roughly every 8 months (Ho et al., 2024). Recent work distinguishing between “compute-dependent” and “compute-independent” algorithmic advances (Sanderson et al., 2025) suggests that the latter, while historically less impactful, could become increasingly significant if scaling encounters limits.

Compounding these trends is the emergence of self-improving coding agents (Huckins, 2025)—LLM-based systems capable of designing and iteratively refining algorithms themselves such as the Darwin Gödel Machine (Zhang et al., 2025) and Google’s AlphaEvolve (Novikov et al., 2025)—which raise new safety concerns.

While only a limited number of frontier models exist (Epoch AI identifies roughly 30 “foundational” models trained above the 10^25^ FLOP threshold, all released after March 2023), the downstream dissemination of AI technologies is vast and increasingly difficult to track. Open platforms such as Hugging Face now host over two million machine-learning models (Raoul, 2024), alongside hundreds of thousands of datasets and applications, supported by a community exceeding five million users (Laufer et al., 2025). Although these models differ fundamentally from the “notable” or foundational models tracked by Epoch AI and often consist of fine-tunes, forks, or task-specific derivatives, the scale of their diffusion illustrates how advances originating from a small number of frontier systems rapidly propagate throughout a pervasive and expanding AI ecosystem.

Facing the rapid improvement of leading models, the widening access through open-source models and the increasing deployment driven by private actors operating across jurisdictions, the task of defining, measuring and ensuring AI safety cannot be left solely to individual governments or to the private sector itself, or just ignored, relying on the belief that the current mainstream development track (based on LLMs) cannot lead to Artificial General Intelligence (AGI) and existential risks (Khalili, 2026; Narayanan and Kapoor, 2025). This raises a critical question: if existing intergovernmental and standards-development mechanisms are structurally constrained, who can credibly take on the task of advancing AI safety science and technologies and of developing sound frontier AI safety standards, and how?

## 4 The case for science-driven international cooperation in AI safety

### 4.1 A shared scientific understanding and focused collaboration

In light of the above considerations, we argue for a science-driven international cooperation in AI safety. A first and fundamental task is to reach a shared scientific understanding of the two sides of the same coin: risk and safety. Over the past decade, a wide range of stakeholders, including researchers, domain experts, civil society representatives and policymakers, have increasingly recognized that advanced AI systems pose risks to individuals and groups, societies and, potentially, to humanity as a whole. Differentiations among types and levels of risk have informed regulatory and governance initiatives such as the EU AI Act (EU, 2024) and the NIST AI Risk Management Framework (NIST AI RMF 1.0, 2021; Autio et al., 2024). Nevertheless, despite repeated warnings from prominent scientists about the possibility of extreme or catastrophic risks (Bengio et al., 2023; Grey and Segerie, 2025), existing frameworks are far from able to address such risks.

Growing awareness within the scientific community about AI risks, combined with the phenomenal advancement of technology, models and systems, have gradually led to the emergence of the AI Safety field (Toner and Acharya, 2022; Gyevnar and Kasirzadeh, 2025; Grey et al., 2025), which is concerned with identifying, evaluating and mitigating harms arising from AI systems across their lifecycle.

While a shared, formal definition of AI safety (from a standardizing body or an international organization) is still missing, the US AI Safety Institute (AISI), prior to its institutional transformation, characterized AI safety as encompassing system reliability and interpretability, as well as evaluation and mitigation of existing, emerging and potential risks to individual rights, public safety and national security (NIST, 2024). Its three strategic goals—advancing the science of AI safety, articulating and disseminating safety practices, and supporting institutional and international coordination—explicitly acknowledged the field’s nascent state. AISI identified core challenges including the absence of commonly accepted definitions and metrics for AI safety capabilities, underdeveloped testing and evaluation methods, a lack of scientifically grounded mitigation strategies across the AI lifecycle, limited understanding of the relationship between model design and behavior, and fragmented coordination among industry, civil society and national and international actors. Notably, the institute was restructured in June 2025 into the Center for AI Standards and Innovation (CAISI), with a substantially altered mandate that no longer includes AI safety (Lutnick, 2025).

Against this backdrop, science-led international efforts outside formal intergovernmental processes have begun to play an increasingly important role. For example, since 2023 the International Dialogues on AI Safety (IDAIS) brings together leading scientists from around the world to collaborate on mitigating AI risks (International Dialogues on AI Safety, 2023). This high-level forum has stressed the importance of global collaboration to address AI risks, highlighting strategic directions and fundamental “red lines” to be considered, although its main message is directed to world leaders, urging them to take action until this is possible.

The International AI Safety Report (IAISR) published in January 2025 (and updated in October and November 2025) presents itself as “the world’s first comprehensive review of the latest science on the capabilities and risks of general-purpose AI systems. Written by over 100 independent experts and led by Turing Award winner Yoshua Bengio, it represents the largest international collaboration on AI safety research to date” (International AI Safety Report, 2025). Despite its great importance, the primary purpose of the report is “serving as the authoritative reference for governments and organizations developing AI policies worldwide,” rather than coordinating scientific activities (ibid).

The Singapore Conference on AI (SCAI), held in 2025 (The Singapore Consensus, 2025), brought together approximately 100 leading AI experts from multiple countries with the explicit aim of advancing technically grounded discussions on AI safety. The conference produced the Singapore Consensus on Global AI Safety Research Priorities, a document that, building on the IAISR, identifies a focused set of priorities across three domains: (1) challenges in assessing and evaluating risks, (2) challenges in the development of trustworthy AI systems and (3) challenges in monitoring and intervention after deployment. This was another important step: compared with broader and mostly policy-oriented reports, the Singapore Consensus, while remaining a general orientation document that outlines promising research directions that laboratories and institutions might wish to undertake, at least in principle, offers a more targeted and actionable research agenda that could be used as a basis to guide concrete scientific collaboration on AI safety.

These high-profile documents provide extensive references to AI safety research directions, and there are many other academic works describing valuable approaches for machine/deep learning systems’ safety. Here we include a few references concerning significant works addressing Taxonomy of Harms, AI Risk Measurement, and Metric for Safe AI (Abercrombie et al., 2024; Giudici et al., 2024; Babaei et al., 2025; Giudici and Kolesnikov, 2026), although a comprehensive survey of this dynamic field is out of the scope of this paper, and should be undertaken in future studies.

Building on and advancing these initiatives, we claim that under the current geopolitical conditions, coordinated, science-led research represents the most viable approach to addressing the risks posed by advanced AI systems and to advancing AI safety. The historical success (at least to a degree) of intergovernmental organizations or agreements in governing science and technology related risks such as the International Atomic Energy Agency (IAEA) and the Biological Weapons Convention (BWC), might make it difficult to imagine effective alternatives.

Yet history offers numerous examples in which research communities sustained transnational networks for knowledge exchange and collective problem-solving, bypassing geopolitical rivalry and industrial competition. We provide a few inspiring examples in what follows—recalling that none of them can be taken literally as a model. However, despite differences of context, actors, scopes and objectives, all examples show that independent entities, involving outstanding, highly motivated individuals and communities, even with limited resources, can enormously increase the collective ability to achieve far-reaching goals through collaboration. This is somehow recognized even by the leaders of top companies engaged in the “AI-race,” when they say that the best minds in the field would be able to “solve” the problem of AI safety, if only they could work together (Hassabis and Amodei, 2026). Unfortunately, they do not want to or cannot take any concrete action in this direction.

### 4.2 Historical examples of science-driven international projects that have achieved far-reaching goals

A paradigmatic example of science-driven collaboration is the Human Genome Project (HGP). Launched in October 1990 and completed in April 2003, it is considered one of the greatest scientific accomplishments in history (Britannica, 2025). The HGP was undertaken by an international group of researchers, looking to comprehensively study all of the DNA (the “genome”) of a select set of organisms and, in particular, to determine the base pairs that make up human DNA, and to identify, map and sequence all of the genes of the human genome.

US scientists played a fundamental role in conceiving and launching the project, and the US National Institutes of Health was the first and probably the most important funding agency of the project. However, it was a fully international project conducted by a network of 20 independent research institutes from the USA, UK, Japan, France, Germany and China that worked together (establishing the International Human Genome Sequencing Consortium, IHGSC) and followed a voluntary, international cooperation agreement based on the so-called Bermuda Principles (Maxson Jones et al., 2018), summarized below:

- The immediate release of any DNA sequence of more than 1.000 base pairs within 24 h from being generated;
- The deposition of data in public databases [GenBank (USA), EMBL (Europe), and DDBJ (Japan)] as soon as possible, with free access for anybody;
- The non-patentability of the raw human genome sequence data, because the genome was considered to be a shared resource of humanity;
- Equal access: no group had privileged access to the data; all scientists worldwide—academic or commercial—could use the data for research, medicine and education;
- Rapid release (before publication): data had to be released before scientific papers were published (researchers could not delay the sharing of data to seek priority).

All participants agreed to follow the Bermuda Principles and to adopt the same standards.

In short, under the Bermuda Principles, HGP participants agreed to release human genome sequence data freely, publicly and as soon as possible, without patent restrictions. There was no single “owner” of the HGP. It was operated as a distributed system, governed by a research community following scientific consensus, through voluntary agreements binding participants and donors. Data were free and access unrestricted.

The HGP achievement was phenomenal and largely unexpected. Its success was particularly remarkable considering the (relatively) limited resources available to the participants, compared to those of the competing private-sector player (Celera Genomics) that was backed by venture capitalists who were interested in patenting results to provide data only to paying customers.

Another important and relevant example concerns the development of the internet, probably the greatest accomplishment of a “bottom-up” process involving a very large, open community of experts and users. Although its initial steps took place in the US with funding by ARPA (the Advanced Research Project Agency, a US government agency created in 1958 to facilitate research in technology with potential military applications, renamed DARPA in 1972), its development and broad dissemination were determined by a global, bottom-up process driven by research and academic institutes and computer experts (Leiner et al., 1997).

The internet is based on the concept of packet switching, a method to move data in distinct, small blocks (packets) over a digital network, routed on the basis of the destination address contained in each packet, and invented in the 1960s independently by Leonard Kleinrock (MIT, USA), Paul Baran (RAND, USA) and Donald Davis (NPL, UK). Packet switching represented a radical innovation compared to the “circuit switching” networks of telephone companies: exchange of data among computers was realized at the time by using dedicated telephone lines, on the top of which ran proprietary communication protocols that were different for each computer vendor. The advantage of packet switching, if properly implemented, is immense, as forecast by its visionary inventors. In short, it allows a much more efficient use of existing networks to manage the exchange of digital data.

In the late 1960s, ARPA funded the development of the Advanced Research Projects Agency Network (ARPANET), the computer network forerunner of the internet, designed to link computers at selected research institutions over telephone lines. The experimental ARPANET grew significantly during the 1970s creating a network of connected research centers and universities, initially in the USA, then in Europe and beyond. The rapid evolution of ARPANET brought to evidence the need for “open standards” that could support software implementations, at various levels, enabling the interconnection of different computers.

To make a long story short, the most important ones were developed by Vinton Cerf and Robert Khan in 1973 (Transfer Control Protocol, TCP, i.e., a protocol for host-to-host internetworking) and in 1978 (Internet Protocol, IP, responsible for packet routing). A few years later, in 1983, ARPANET was divided in two: the civilian part, called ARPA Internet and the military part, called MILNET (this event is marked as the “big bang” of the internet). At the same time, ARPA decided to put the technology in the public domain and “the Internet Activities Board (IAB)” was established. As indicated in the (now historical) RFC 1120, *“the IAB is the coordinating committee for Internet design, engineering and management. […] The IAB is an independent committee of researchers and professionals with a technical interest in the health and evolution of the Internet system. The IAB focuses on the TCP/IP protocol suite, and extensions to the Internet system to support multiple protocol suites* (Cerf, 1989).

The advent of the personal computer in the 1980s and the set-up of Local Area Networks based on TCP/IP (such as Ethernet) connecting PCs and other computers in offices, gave tremendous momentum to the dissemination of TCP/IP and the growth of the internet. By the early 1990s the process was fully consolidated: to give an idea, in Europe there were 30,000 internet sites in 1990, and they grew to 100,000 in 1992. All this happened before the invention of the World Wide Web, considered the “killer application” of the internet, and the release of the WWW source code in the public domain by CERN in 1993. After that the internet “exploded,” bringing the modern era of digital communications (The development of the WWW, its dissemination and the establishment of the W3C consortium responsible for the governance of the Web, is another outstanding example of the power of collaboration).

What is particularly important, from our perspective, is that this process was entirely bottom-up, driven by research and academic institutes and computer experts, before reaching the business community and the general public. The development of the technologies and governance of the internet was performed by committees involving “researchers and professionals with a technical interest in the health and evolution of the Internet system,*”* through structures set up after 1983, that today are part of the internet ecosystem, defined together as the “Internet Society” (Internet Society, 2026).

It is also instructive to highlight that for some years (mid 1980s–early 1990s), especially in Europe, the internet “movement” encountered significant resistance, especially from governments, public institutions and big business. Strictly speaking, the internet was illegal: telecommunications were operated by monopolies, either government-owned national telephone and telegraph companies, or regulated private companies. In addition, technical competition was *expected from the implementation* of standards from the ISO-OSI (Open System Interconnect) effort—supported by the leading telecommunication and computer companies. However, for a variety of reasons, including the inertia of telecommunication companies in abandoning their established business models, and the reluctance of leading computer manufacturers to hinder their proprietary networking solutions, such implementations did not materialize in time.

A third outstanding example concerns the Global Observing System (GOS); the system that provides observations on the state of the atmosphere and ocean surface—data used for the preparation of weather analyses, forecasts, advisories and warnings, for climate monitoring and more (GOS, 2023). The GOS is one of the most ambitious and successful instances of international collaboration of the last 100 years. It consists of thousands of individual observing systems (terrestrial, maritime, aerial and satellite-based), owned and operated by a plethora of national and international entities. And yet the overall requirements and reporting practices (formulated by bodies belonging to the World Meteorological Organization, WMO^3^) are almost universally adopted. As indicated by the WMO, “*Since the establishment of the World Weather Watch in 1963, the Global Observing System (GOS) has been the major mechanism for providing continuous and reliable observational data worldwide*.” (ibid). Although the GOS is a system conceived and governed in the framework of an intergovernmental organization under the UN system—the WMO—the reason why we mention it here relates to the phenomenal achievement derived from the scientific and technical collaboration of a myriad of independent actors.

The International Network of AI Safety Institutes provides a different example of an attempt to establish international collaboration on AI safety. Proposed by two main bodies (UK and US AISI) in 2024 and conceived as an informal, closed network of AISIs, at the AI summit in Seoul (May 2024) it was endorsed by the governmental bodies of 11 countries, including countries where an AISI was already operational and others that intended to establish it. This network was aspirational, with the primary objective of exchanging knowledge and results on the topics that each AISI was addressing—however, given the changes in orientation and the geopolitical context described above, this network is hardly operational today.

### 4.3 The proposed network for AI safety builds on existing initiatives but goes farther

The Network proposed in this article should be a larger, open entity, involving all relevant actors (AI labs, research institutes, think tanks, AISIs and more) that have the capabilities and interests in pursuing the Network’s mission and goals, as highlighted in the first section (“our proposal”). Elements that need to be agreed, reviewed and regularly updated by the constituency.

We recognize that existing initiatives that articulate promising research agendas such as the International AI Safety Report and the Singapore Consensus represent important steps forward. However, despite the scarcity of resources devoted to AI safety research, current efforts remain largely fragmented, with research being undertaken by individual organizations and with limited levels of sustained exchange. Some institutions have sought to aggregate external contributions by inviting others to participate in their own programs and to follow their agendas. While laudable, such approaches fall short of what is required to achieve meaningful progress within the narrow time horizon imposed by rapidly advancing AI capabilities.

As we have outlined in the previous section, history suggests that bringing together a plurality of research entities, under a genuinely shared agenda through voluntary cooperation, is not only possible but essential. Without such a concerted, science-led effort, the prospects for developing robust and timely AI safety solutions remain severely constrained. However, we recognize that the proposed initiative is undoubtedly ambitious, and at least three main challenges need to be addressed.

### 4.4 Key challenges to be addressed

Firstly, what are the incentives to join the initiative?

- We address this essential question from two sides: supply (the incentive to join and contribute) and demand (the incentive to use the Network’s deliverables). It is well documented that many of the world’s leading scientists and experts are genuinely and strongly concerned about the current risks of AI (Pacing the Frontier, 2026; Egan, 2026; Bove, 2026; Bengio, 2025b). Many worry that the discipline they have contributed to create and develop may harm people and society, including their children and grandchildren. This awareness is the driving force behind their commitment and public engagement, in many forms and forums. Joining the Network will give them the opportunity to take a step forward, contributing in concrete terms to address some of the most formidable global challenges. This also concerns young people, including many of the most promising talents in the field. Despite the lack of general survey, emerging career-consulting non-profit organizations such as Probably Good^4^ and 80,000 h^5^ indicate that many young AI professionals would pursue career providing rewards in terms of contributing to problems and breakthroughs that are impactful and “good” for the world. The hope that “Linus Torvalds” or “Tim Berners-Lees” of AI safety could emerge from such a network is not merely wishful thinking; it is a plausible outcome of giving talented, mission-driven people a credible platform to tackle the problem.

On the demand side, the case is less about vision and goodwill and more about the core driver behind timely and successful deployment of applications. AI developers operate under intense commercial and competitive pressure, and safety work that generates additional burdens and delays, or performance trade-offs, is often deprioritized. The Network’s role, then, is to change that calculus. If the Network can offer deliverables (safety tools and guidance on how to use them) that are open, high-quality, interoperable, efficient and cheap to integrate—ideally lowering rather than raising development costs—adoption stops being a matter of burden and becomes a matter of self-interest. Furthermore, at some point the Network’s deliverables may well be endorsed, recommended or even required by regulators in various jurisdictions. AI developers that already use them would find themselves already in compliance, without extra effort. This is a mechanism that, in different fields, has often triggered the early adoption of standards and deliverables based on them, on the expectation that those would be later incorporated into regulatory requirements.

The second foreseeable challenge regards selfishness

- There is often a strong tendency for leading scientists and experts, along with the research teams they lead or influence, to pursue their own agendas, shaped by their particular expertise, institutional incentives, and deep-rooted convictions. Compounding this, participants may have or perceive significant conflicts of interest: suspicion about the real commitment of other members, and concerns that shared ideas or methods could be appropriated by others, leading to undue recognition or unfair economic exploitation. Together, these dynamics substantially raise the difficulty of designing and operating an adequate governance structure. Yet the history of science suggests this tension is not fatal: fierce competition and genuine collaboration have coexisted and even reinforced each other in past scientific endeavors (Daston, 2023) and there is no structural reason the same could not hold for an AI safety network, provided its governance is designed with this tension in mind from the outset. The common overarching goal should be well worth some compromises on the strategic directions and the choice of specific technical approaches. The Bermuda principles and the approach followed for the development of the internet, as mentioned in previous sections, can provide useful inspiration and insights.

Lastly, there are the challenges of funding the network’s research and development activities.

- How to secure sufficient and sustained funding, and how to share and balance resources and contributions fairly among the various participating entities? On securing funding, history offers durable rationales that have reliably mobilized both public and private resources for large-scale scientific cooperation, even absent short-term commercial payoffs: prestige, peace and challenges that are civilizational in scale and require shared, rather than competitive, investment—for examples, in fields such as astronomy (Chinnici, 2022), nuclear security (IAEA, 2024), and climate change (IPCC, 2024).

AI safety fits this pattern naturally: the risks under discussion are explicitly transnational, and establishing the Network as an instrument of collective security offers a peace-and-security rationale for funding that sits alongside and may prove more durable than purely scientific or commercial justifications.

As previously outlined, the proposed approach focuses on seeking mission-aligned funding from private and non-profit foundations, philanthropies and other organizations with established interests in global risks, and in AI safety, ethics, alignment and related areas. Funding from states would be welcome, although it should not alter the science-based, autonomous process set up for identifying research priorities and implementation. It is plausible that “middle powers,” states lacking resources or ambitions to compete on frontier AI capability development, but interested in acquiring meaningful influence, visibility, and diplomatic standing, might wish to support the Network as an instrument for collective good.

The question of how resources and contributions should be balanced among participating entities is a fundamental governance matter requiring specific mechanisms to manage in-kind contributions (compute, data, and personnel) alongside direct funding, and transparent criteria for the allocation of resources in line with research priorities. Addressing these questions requires dialogue, constructive negotiation and above all trust among prospective members and funders. It is precisely this process that the ideas set out in this paper hope to stimulate.

## 5 Recommendation and discussion

### 5.1 Recommendation

This paper is intended as a call to action primarily directed to the scientific community worldwide involved in AI research and development. It advocates for their proactive and concrete engagement, and for joining forces to create a scientific and technical framework enabling the development of safe(r) AI systems.

We call for science-driven international cooperation in frontier AI safety governance through the creation of a dedicated international network or consortium focused on AI safety (the Network), run by leading scientists from relevant disciplines (involving the research entities where they work) and operating independently of formal national affiliations.

The Network should pursue three strategic goals.

First, advance and coordinate AI safety science. Participants in the Network should undertake coordinated research aimed at advancing the scientific foundations of the emerging field of AI safety.

Second, develop practical techniques, tools, best practices, and technologies for designing and building safe(r) AI systems—building on, and in parallel with advances in AI safety science.

Third, develop standards and conformity assessment mechanisms for safe(r) AI. The Network should translate the outputs of Goals 1 and 2 into draft standards that operationalize AI safety in practice, including specifications, documentation of procedures and good practices, criteria and thresholds, and test methods—potentially supported by AI-enabled software suites. We underline that these deliverables would function as “pre-standards,” to be submitted, in due course and through mechanisms to be established, to formal international standardization bodies for review, refinement and possible adoption.

More information on the Network and a possible implementation strategy has been presented under Section 2 and considerations on the challenges to be addressed, under Section 4.4.

### 5.2 Discussion

Along with the challenges regarding the implementation of the recommendation, we acknowledge that there may be objections to the rationale of the proposal, and requests for a more detailed and analytical assessment of some of our assumptions—for example that in the current context ongoing multilateral initiatives are seriously constraint and insufficient to satisfactorily mitigate the most severe AI risks.

However, we are convinced that the level of risks and its rapid increase, and the lack of common understanding and action from governments and policy makers, highlighted almost daily by mainstream media (Sanger and Volz, 2026), require conceiving and promoting significant and concrete actions as soon as possible. In this respect a remarkable example is Yoshua Bengio’s LawZero (Bengio, 2025a; Bengio et al., 2026) and we hope that the Network proposed in this paper could be another one.

In addition, even admitting by absurdity that some of our assumptions are incorrect, we cannot see any downside to the proposal. A coordinated, focused effort of some of the best researchers to advance AI safety, can only be beneficial to any initiative in the field.

As outlined in the proposal, the International Association for Safe and Ethical AI (IASEAI), set up in 2024 “to unite the efforts of hundreds of organizations and thousands of individuals concerned with safe and ethical AI”, could play, in this respect, a very important role, IASEAI already engages many leading scientists advocating action for AI safety, including notably its President Russell (2022) and Russell (2019), Board member Yao (2024), Advisory Board members Crawford (2021), Tegmark and Omohundro (2023), and Dalrymple et al. (2024). It is possible to see IASEAI contributing to the offspring, from the community they represent (now mostly dedicated to advocacy and information sharing), of a project-based, outcome-oriented entity.

### 5.3 Indications for future research

Finally, there are many subjects that require additional research efforts to support and accompany the proposal. A preliminary outline is given below.

First and foremost, a refinement of the proposal: with more detailed analysis and description of its possible operationalization (considering aspects such as membership, governance, safeguards and funding) and a sound draft project plan for its implementation.

It would also be important to analyze in detail existing literature and efforts concerning the classification and quantification of risks posed by existing and possible future AI systems—an activity that could be conducted prior to the launch of the Network or in the framework of its work programme.

Specific efforts should be dedicated to operate a preliminary selection of promising key approaches aimed at enabling “safe by design” AI systems—relying on reference publications and processes (such as the International AI Safety report, proceedings of the Independent International Scientific Panel on AI, the Singapore Consensus) and on original, focused efforts of some of the parties interested in building the Network.

Another particularly important subject area concerns “Benchmarking AI safety”. A very broad field covered independently by many actors, which should be structured across different domains, considering a variety of approaches and techniques, with a view to proposing actionable patterns to the Network.

Last but not least, studies in the emerging field dealing with countermeasures to contrast “rogue” AI behavior, would certainly be desirable.

## Footnotes

1 Requirements for GPAIs are primarily technology-based, differently from the overall AI Act approach that is application and user-based: an aspect that has raised many questions and challenges. In addition, the complexity of requirements considered for GPAIs (especially for those posing a “systemic risk”) and the issues regarding their implementation and real effectiveness, are extensively debated. A detailed analysis of these subjects goes beyond the scope of this work: however, just to give an idea, consider that Open AI–with all the safety concerns that its models increasingly pose—claims that its safety practices are fully aligned with those required by the AI Act (Daws, 2026).

2 Legally binding rules in China include Primary Laws, promulgated by the National People’s Congress (NPC), the national legislature of the People’s Republic of China, and Administrative regulations and departmental rules, issued by the State Council and its ministries. The latter typically provide detailed descriptions and implementation guidance of primary laws, or interim provisions when no primary law exists.

3 Interestingly, the WMO itself has evolved from the International Meteorological Organization (IMO), founded in 1873 as a voluntary association whose members–directors of national meteorological services–participated in their personal capacity as scientists rather than as representatives of sovereign states. Through its technical commissions, the IMO achieved substantial standardization of meteorological observation, including instruments, timing, formats and reporting practices, and expanded global observing networks across periods of imperial expansion and through the two world wars and the Cold War.

4
https://probablygood.org/about/

5
https://80000hours.org/about/
